# How do price spillovers between natural gas and copper-aluminum markets differ in international vs. Chinese contexts?

**DOI:** 10.1371/journal.pone.0345129

**Published:** 2026-04-02

**Authors:** Lei Wang, Qingpeng Lu, Tingqiang Chen, Binqing Xiao

**Affiliations:** 1 School of Economics and Management, Nanjing Tech University, Nanjing, China; 2 School of Management and Engineering, Nanjing University, Nanjing, China; Beihang University, CHINA

## Abstract

To investigate time-varying spillover effects among the natural gas, copper, and aluminum markets and to compare heterogeneity between the Chinese and international markets, this paper constructs a DGC-t-MSV model. Based on Bayesian estimation and MCMC sampling, the model captures time-varying conditional volatilities and dynamic correlations, thereby quantifying bidirectional spillovers across markets. Research demonstrates that: (1) Two-way price spillovers exist between the international and Chinese natural gas, copper, and aluminum markets. Spillovers between international natural gas and metals are more pronounced. Price spillovers in these markets are not constant over time and exhibit asymmetry in various contexts. (2) Price spillovers between the international and Chinese natural gas and aluminum markets are significant. However, the correlation between natural gas and copper is even stronger. One-way spillover effects suggest that the copper and aluminum markets act as risk absorber. (3) Price spillover effects are more pronounced from the international natural gas market to the Chinese copper and aluminum markets, especially for aluminum. In mature markets, natural gas, copper, and aluminum exhibit lower volatility. However, spillover effects intensify under extreme risk conditions.

## 1 Introduction

The stability of the natural gas, copper, and aluminum markets is essential not only for industrial development and quality of life, but also for ensuring employment stability. However, recent geopolitical risks, particularly the Russia-Ukraine war, have significantly affected these markets, raising concerns about international supply instability. Natural gas is essential in the smelting and processing of copper and aluminum. Its price fluctuations directly impact production costs and, through the tariff mechanism, can significantly influence the power consumption costs of smelting enterprises [1,2]. Natural gas remains critical in copper and aluminum smelting. However, technological advancements in smelting processes may reduce this dependence [3,4]. Such shifts could alter natural gas price volatility. These dynamics increase operational risks for industries and threaten market stability. Therefore, studying the effect of changes in prices between the markets for natural gas, copper, and aluminum is important. The study provides insights into their interdependencies. It also informs strategies to stabilize economic development amid evolving energy and industrial landscapes.

Research on energy-metal market spillovers has attracted growing scholarly interest. Existing research focuses on three dimensions. First, some studies investigate volatility transmission among energy subsectors, including conventional energy, renewable energy, and metals [3,5]. For example, Chen et al. demonstrate the hedging effectiveness of non-ferrous metals for clean energy assets across different time-frequency domains. Li et al. identify renewable energy as a dominant risk transmitter in integrated energy-metal systems. Second, spillover effects propagate through third-party markets. The electricity market is a persistent spillover receiver in energy-metal networks [6]. Agricultural markets also exhibit sensitivity to energy and metal price fluctuations [7,8]. Third, external shocks critically reshape spillover mechanisms. Policy shifts and geopolitical events generate asymmetric transmission patterns [9]. Climate policy uncertainty exerts heterogeneous impacts on energy-metal spillovers [2]. While existing research on price spillovers between energy and metal markets is a common focus in most studies. There is a lack of detailed analysis regarding natural gas price volatility with copper and aluminum markets. This gap complicates the understanding of spillovers between natural gas and these metals. Therefore, this study seeks to analyze the effects of price spillovers among the natural gas, copper, and aluminum markets, considering market segmentation.

Scholars have used the GC-MSV and DCC-MSV models to research price spillovers among multiple markets [10,11]. However, the GC-MSV model is relatively complex in parameter estimation, has strict requirements on the quality and quantity of data, and may be limited by model assumptions. The shortcomings of the DCC-MSV model are that its model construction is relatively complex, and the results are susceptible to the selection and adjustment of parameters. To compensate for these shortcomings, scholars have begun to explore other extended portfolio models. For instance, Wang et al. applied the DGC-t-MSV model to analyze cross-market risk spillovers. Their findings suggest that the model is more effective in capturing key information, such as correlations and volatility heterogeneity within market dynamics. Additionally, it demonstrates greater flexibility when modeling extreme events. Inspired by this, the DGC-t-MSV model is chosen in this paper to study the two-way price spillover effects among the natural gas, copper, and aluminum markets.

In summary, this paper addresses the shortcomings of existing research by considering the time dynamics of price spillover effects. It expands the DGC-t-MSV model by introducing the dynamic conditional correlation coefficient and Granger causality test. The Bayesian method and MCMC technology are used to estimate model parameters, analyzing price spillovers among the international and Chinese natural gas, copper, and aluminum markets. Innovative points of this article: (1) Unlike prior studies that took a broad view of energy and metals, this paper develops a unified comparative framework for the natural gas, copper, and aluminum markets by jointly examining the international market and the Chinese market within the same empirical design, which allows a clearer identification of cross-market and cross-region differences under market segmentation. (2) The study Integrates the DGC-t-MSV model with Bayesian and MCMC methods. Even with short datasets, it incorporates time-varying factors to efficiently measure bidirectional spillover effects among natural gas, copper, and aluminum markets. In particular, it captures time-varying conditional volatilities and dynamic correlations, allowing spillover intensity to be evaluated across different market regimes. (3) Key findings: Bidirectional price spillover effects exist among the international and Chinese natural gas, copper, and aluminum markets. Spillover effects are time-varying and asymmetric. The most significant spillover effects occur from the international gas market to the international copper and aluminum markets, as well as from the Chinese gas market to the Chinese copper and aluminum markets. Price spillovers between the natural gas and aluminum markets are more significant than those between the natural gas and copper markets. However, the correlation between the natural gas and copper markets is more substantial in the international and Chinese markets.

The paper is structured as follows: the second part constructs the DGC-t-MSV model. The third part describes the estimation of the model parameters and analyses the price volatility spillovers among the natural gas, copper, and aluminum markets. The fourth part draws the main conclusions of the paper.

## 2 Theoretical transmission mechanism

This subsection outlines the theoretical channels through which shocks to natural gas prices spill over to copper and aluminum markets. This paper identifies four primary mechanisms: the demand and macro-expectations channel, the financialization and risk-spillover channel, heterogeneous production technologies across metals, and institutional differences between international and Chinese markets. The detailed analysis is presented below:

First, the demand and macro-expectations channel. Natural gas prices reflect not only their own supply–demand conditions but also broader macroeconomic information, including global business-cycle dynamics and extreme weather shocks [12–14]. A sharp increase in natural gas prices is typically accompanied by higher energy expenditures for households and firms, a squeeze on real income, and weaker expectations regarding industrial production and investment, which jointly reduce final demand for base metals such as copper and aluminum. From an asset-pricing perspective, when higher natural gas prices are interpreted as a combination of cost-push inflation and weakening aggregate demand, copper and aluminum prices are jointly affected by rising production costs and deteriorating demand conditions, altering both their levels and volatilities.

Second is the financialization and risk-spillover channel. Natural gas and base-metal futures are now widely traded financial assets and are included in many commodity indices and multi-asset portfolios. Large moves in natural gas prices can change investors’ risk appetite and tighten margin constraints, leading them to rebalance positions across different contracts and markets. When indicators of geopolitical risk and market uncertainty, such as geopolitical risk indices or volatility indices, increase, investors often cut their exposure to high-risk energy and metal futures at the same time, or take offsetting positions in other contracts to limit possible losses. These trading actions create clear comovements and volatility spillovers between natural gas and metal prices [15–17], transmitting price shocks across commodities and markets through shifts in risk preferences and asset-allocation decisions.

Third, heterogeneous spillovers between copper and aluminum. Because production technologies and energy-use structures differ, the impact of natural gas prices on copper and aluminum is asymmetric [18,19]. Aluminum smelting is highly electricity-intensive, and electricity accounts for a large share of total production costs. In many European and North American economies, natural gas represents an important share of power generation, so shocks to natural gas prices are readily transmitted to aluminum’s marginal production cost through higher electricity prices, leading to stronger effects on aluminum prices and volatility. In China, by contrast, regulated electricity tariffs can limit this pass-through. Copper smelting is relatively less dependent on electricity, and copper prices are more sensitive to global manufacturing activity, infrastructure investment, and speculative and hedging demand, exhibiting a stronger financial character. Under comparable natural gas shocks, aluminum prices therefore display a more pronounced cost-driven pattern, whereas copper prices are more strongly driven by macroeconomic and financial factors.

Fourth, institutional differences between international and Chinese markets. The mechanism diagram highlights cross-regional differences in institutional settings and energy structures. In many international markets, especially in Europe and the United States, natural gas accounts for a large share of power generation and energy prices are largely market-determined, so changes in natural gas prices are transmitted directly to local copper and aluminum prices through electricity prices and smelting costs. In China, by contrast, aluminum smelting relies mainly on coal-fired power, natural gas represents only a small share of the power mix, and domestic gas prices, electricity tariffs, and the smelting industry are subject to strong policy interventions such as electricity price caps, energy subsidies, capacity-control measures, and carbon-emission constraints [20,21]. Consequently, the cost-based transmission chain from international natural gas markets to Chinese copper and aluminum markets is weak, while spillovers through policy expectations, import costs, and exchange-rate movements are more important; these institutional and energy-structure differences jointly determine the direction and magnitude of the cross-market and cross-metal price spillovers documented in the empirical analysis. This buffering role of regulated electricity prices provides a direct explanation for our empirical finding that spillovers are weaker in China than in international markets, because natural gas volatility is less likely to be fully transmitted into domestic aluminum production costs.

**Fig 1:**
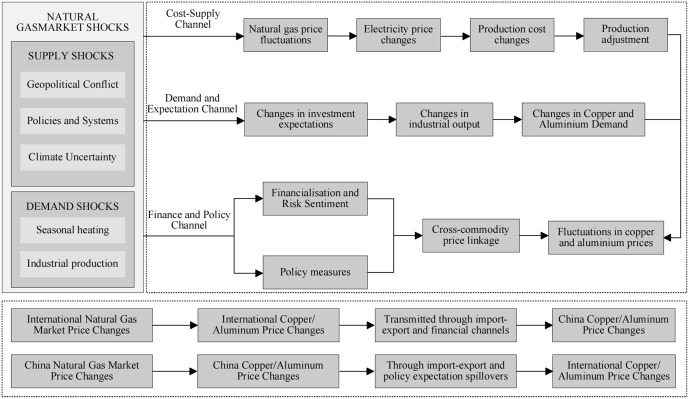
Mechanism diagram of price fluctuations between natural gas and copper-aluminum markets.

Building on the above discussion, Fig 1 summarizes the main channels through which natural gas price shocks transmit to copper and aluminum prices and volatility.

## 3 Model construction

This study employs a multivariate stochastic volatility (MSV) model and its dynamic extension, which analyzes dynamic correlations and volatility spillovers among the international and Chinese natural gas, copper, and aluminum markets. The MSV framework simultaneously captures multivariable interdependencies and time-varying fluctuation dynamics [22,23]. The extended MSV model integrates Dynamic Generalized Correlation (DGC) and Multivariate Stochastic Volatility (MSV) to capture time-varying correlations. It accounts for spiky, thick-tailed features in financial time series, enhancing its ability to handle non-normal data [24–26].

### 3.1 Data sources and pre-processing

In this article, the international natural gas, copper, and aluminum data are sourced from Invesco Finance. They are taken from the closing futures prices from 1 January 2022–31 October 2024 on the New York Stock Exchange in the US. The Chinese natural gas data is sourced from Wind Database. Since there is no domestic natural gas futures data in China, this paper uses the closing price of New Natural’s stock from 1 January 2022–31 October 2024 to conduct a price volatility spillover analysis. The Chinese copper and aluminum data are sourced from Wind Database, and their futures closing prices are selected. To guarantee the precision and dependability of the research findings, this paper pre-processes all the collected data, that is, calculating the logarithmic rate of return.

### 3.2 Basic MSV model

Construct a basic MSV model to analyze the market’s yield series:


$$y_{t}=diag\left(exp\left(\frac{q_{t}}{\text{2}}\right)\right)\in_{t},\in_{t}\sim \;N\left(0,R_{t}\right)$$ (1)

In Equation (1), the parameter$y_{t}$ is known to be the yield series of the natural gas market and the copper and aluminum market, $\varepsilon_{t}$ denotes the volatility of the yield series, and$q_{t}$ denotes the market volatility series.


$$q_{t+1}=\mu +diag\left(\phi_{11},\phi_{22}\right)\left(q_{t}-\mu \right)+\xi_{t},\xi_{t}\mathrm{\backslash stackrel}iid\sim \;N\left(0,diag\left(\overset{\text{2}}{\underset{\xi 1}{\sigma }},\overset{\text{2}}{\underset{\xi 2}{\sigma }}\right)\right)$$ (2)

In Equation (2), μ denotes the long-run mean, $\phi_{11}$,$\phi_{22}$ is a parameter describing the persistence of market volatility, $\xi_{t}$ signifies the independent variation in the volatility of the return series, and σ denotes the standard error of the volatility disturbance.

### 3.3 GC-MSV model

In order to analyse the volatility spillovers among the natural gas, copper, and aluminum markets in both international and Chinese contexts, this paper utilizes a generalised conditional multivariate stochastic volatility model (GC-MSV). The model adds an inter-market volatility spillover term to the basic MSV model:


$$\begin{array}{c} q_{t+1}=\mu +\left(\begin{array}{cc} @cc@\phi_{11} & \phi_{12}\\ \phi_{21} & \phi_{22} \end{array}\right)\left(q_{t}-\mu \right)+\xi_{t},\xi_{t}\mathrm{\backslash stackrel}iid\sim \;N\left(0,diag\left(\overset{\text{2}}{\underset{\xi 1}{\sigma }},\overset{\text{2}}{\underset{\xi 2}{\sigma }}\right)\right) \end{array}$$ (3)

In Equation (3), the test is added to the basic MSV model. When the results of $\phi_{12}$ and $\phi_{21}$ are not zero, it means that two series exhibit statistically significant bidirectional volatility spillover effects.

### 3.4 DCC-MSV model

To better understand the dynamic correlation among the international and Chinese natural gas, copper, and aluminum markets, this paper employs a dynamic conditional correlation multivariate stochastic volatility model (DCC-MSV) for analysis. In this model, the correlation coefficients between markets can vary over time rather than just being fixed constants:


$$y_{t}=diag\left(exp\left(\frac{q_{t}}{\text{2}}\right)\right)\in_{t},\in_{t}\sim \;N\left(0,R_{t}\right)$$ (4)

In Equation (4), $R_{t}$ is the correlation matrix over time, which is defined in equation (5):


$$R_{t}=diag{\left(Q_{t}\right)}^{-1}Q_{t}diag{\left(Q_{t}\right)}^{-1}$$ (5)

$Q_{t}$ of the dynamic evolution process in equation (6):


$$Q_{t}=\left(1-\alpha -\beta \right)\overset{―}{Q}+\alpha \in_{t-1}\in_{t-1}+\beta Q_{t-1}$$ (6)

In Equation (6), α and β are the non-negative parameters to be estimated, and \stackrel_Q is the unconditional correlation matrix of the residuals.

### 3.5 DGC-t-MSV model

In order to allow the model to better adapt to complex and changing situations and further increase its flexibility, this paper extends the methodology to a dynamic generalized conditional distribution multivariate stochastic volatility model (DGC-t-MSV). Unlike the DCC-MSV, which typically assumes a conditionally Gaussian joint distribution, the DGC-t-MSV model employed in this study incorporates heavy-tailed Student-t innovations and time-varying stochastic volatility. The Student-t distribution accounts for extreme price movements and leptokurtosis in return distributions, while the MSV framework captures the time-variation in both volatility and correlations. Consequently, this specification is particularly effective for modeling stress periods, such as the Russia–Ukraine conflict, where natural gas and metal markets exhibited sharp price spikes and marked asymmetric dynamics.


$$r_{t+1}=v_{\text{0}}+v_{ac}\left(r_{t}-v_{\text{0}}\right)+\sigma_{\rho }o_{t},o_{t}\sim \;N\left(0,1\right),\rho_{t}=\frac{exp\left(r_{t}\right)-1}{exp\left(r_{t}\right)+1}$$ (7)

In Equation (7), $v_{\text{0}}$ is a parameter describing the dynamic correlation; $\sigma_{\rho }$ is the standard deviation of the noise component; $\rho_{t}$ expresses fluctuating correlation, ranging from −1–1.

### 3.6 Markov monte carlo method and gibbs sampling

In this paper, Bayesian methods and Markov Chain Monte Carlo (MCMC) techniques are used to sample the posterior distributions of parameters in the above models [27]. In MCMC, the Markov chain is defined as:


$$p\left\{X_{\text{0}}=x_{\text{0}},X_{\text{1}}=x_{\text{1}},...,X_{t}=x_{t}\right\}=p\left(X_{\text{0}}=x_{\text{0}}\right)\prod_{t-1}^{t}p\left(X_{t}=x_{t}|X_{t-1}=x_{t-1}\right)$$ (8)

In Equation (8), $p\left(X_{t}=x_{t}|X_{t-1}=x_{t-1}\right)$ denotes the conditional transfer probability of the t th sample over the t−1 th sample. As time t increases, the Markov chain gradually converges to the posterior distribution.

Gibbs sampling is a technique that utilizes the Markov chain Monte Carlo (MCMC) approach and is suitable for generating samples from a high-dimensional joint probability distribution. In the multivariate stochastic volatility model (MSV), Gibbs sampling updates one parameter at a time while keeping the other parameters fixed. Since the posterior distributions of the parameters are usually complex, generating samples from the conditional distributions through Gibbs sampling can effectively estimate the model parameters. The specific procedure is:

(1) Initial setting $\overset{\left(0\right)}{\underset{\text{1}}{\theta }},\overset{\left(0\right)}{\underset{\text{2}}{\theta }},......,\overset{\left(0\right)}{\underset{\text{n}}{\theta }}$.
(2) For the t th iteration:

A: Sampling from conditional distributions $\overset{\left(\text{t}\right)}{\underset{\text{1}}{\theta }}\sim \;p\left(\theta_{\text{1}}|\overset{\left(\text{t-1}\right)}{\underset{\text{2}}{\theta }},...,\overset{\left(\text{t-1}\right)}{\underset{n}{\theta }},y\right)$.

B: Sampling from conditional distributions $\overset{\left(\text{t}\right)}{\underset{\text{2}}{\theta }}\sim \;p\left(\theta_{\text{2}}|\overset{\left(\text{t}\right)}{\underset{\text{1}}{\theta }},\overset{t-1}{\underset{\text{3}}{\theta }},...,\overset{\left(\text{t-1}\right)}{\underset{n}{\theta }},y\right)$.

C: Continue this process until $\overset{\left(\text{t}\right)}{\underset{n}{\theta }}\sim \;p\left(\theta_{n}|\overset{\left(\text{t}\right)}{\underset{\text{1}}{\theta }},...,\overset{\left(\text{t}\right)}{\underset{n-1}{\theta }},y\right)$.

Through continuous iteration, many samples from the target posterior distribution are eventually generated. These samples can be used to estimate the model’s expectation, variance, and other characteristics.

## 4 Analysis of empirical results

In order to show the dynamic correlation results and volatility spillover effects among the international and Chinese natural gas, copper, and aluminum markets prices more intuitively, this paper conducts numerical analyses with the help of MATLAB 2024a software. The datasets generated during the current study are available in the Figshare repository.

### 4.1 Statistical data description

Table 1 presents descriptive statistics for the index returns. The statistics indicate that the skewness and kurtosis values of the index returns are significant, implying that the returns deviate from a normal distribution and instead exhibit sharp peaks and heavy tails. This phenomenon indicates a higher likelihood of extreme price volatility, asymmetric asset price fluctuations, and high-frequency volatility spillovers in the market [28]. External shocks, such as geopolitical conflicts and international policy changes, can amplify the price interconnections between international markets and Chinese markets for natural gas, copper, and aluminum [29,30]. Moreover, since China’s natural gas series is proxied by a listed equity, the return series may incorporate some stock-market fluctuations, which can further contribute to non-normal features in the observed returns. This asymmetry and thick-tailed phenomenon justify using multivariate stochastic volatility (MSV) models. Such complex patterns of volatility, as well as their transmission effects across markets, can be captured more effectively with the help of MSV modeling.

**Table 1:** Descriptive statistics of index returns for international and chinese natural gas, copper, and aluminum markets.

Column	Mean	Median	Maximum	Minimum	Standard Deviation	Skewness	Kurtosis	Jarque-Bera Stat
NG-China	−0.00036	−0.00040	0.10550	−0.09540	0.02266	0.10194	3.73408	436.44507
AL-China	0.00043	0.00091	0.21333	−0.22738	0.04821	−0.22492	3.24662	335.26726
CU-China	−0.00003	−0.00058	0.05727	−0.03859	0.01030	0.77552	3.75101	514.18214
NG	0.00053	0.00153	0.18566	−0.14015	0.03461	0.16097	1.71160	94.66170
AL	−0.00012	−0.00015	0.04530	−0.05298	0.00995	0.04666	3.05590	291.71112
CU	0.00001	−0.00011	0.05338	−0.07299	0.01459	−0.01665	1.32263	54.62913

**Fig 2:**
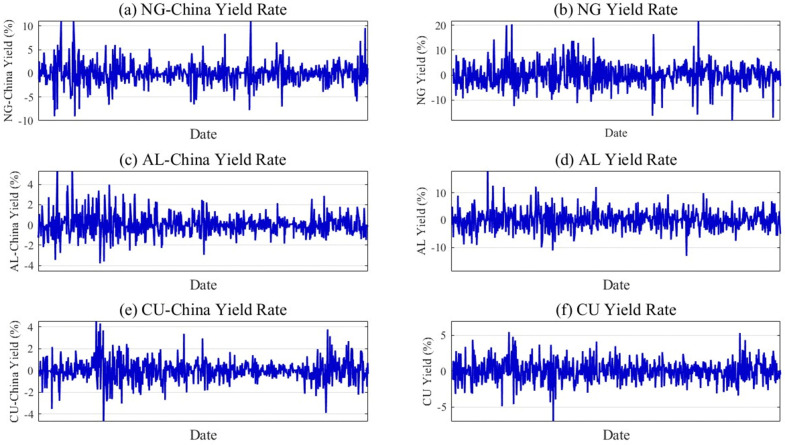
Time series charts of natural gas, copper, and aluminum yields for international and chinese markets.

Fig 2 shows the yield series for the natural gas, copper, and aluminum in both the international and Chinese markets, highlighting the volatility characteristics of each index. The figure shows a clear consistency in the fluctuation frequencies between the Chinese and global natural gas, copper, and aluminum markets. The fluctuations are correlated across these six markets. The international and Chinese natural gas-aluminum markets exhibit bidirectional correlations. Volatility spillover effects also link the international natural gas and copper markets and their Chinese counterparts. This cross-market transmission reflects the influence of natural gas price fluctuations on copper and aluminum markets [31,32]. As a significant energy consumer, the prices of natural gas in China are strongly linked to electricity prices. Shifts in gas prices drive electricity price volatility, directly impacting smelting and production expenses for copper and aluminum. Such interdependencies underscore the systemic risks embedded in energy-intensive industrial systems.

### 4.2 Convergence analysis of the DGC-t-MSV model

**Fig 3:**
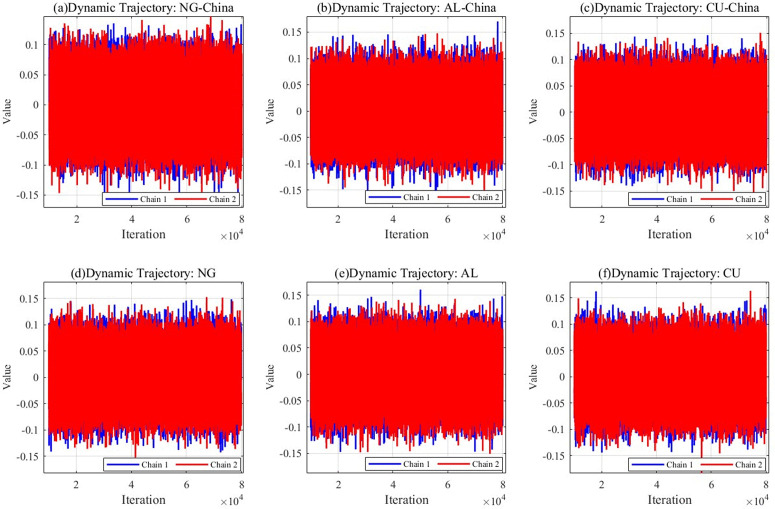
Dynamic trajectories of international and chinese natural gas, copper, and aluminum markets.

In this paper, a Markov Monte Carlo method based on Gibbs sampling is selected to carry out the parameter estimation. Nine sets of index data were analyzed in depth with the help of MATLAB 2024a software. In the parameter estimation session, up to 80,000 iterations are performed, and, as a routine, the initial 10,000 iterations are discarded as warm-up data. This ensures that the subsequent analysis is both accurate and reliable. Fig 3 mainly shows the dynamic tracking results and time series plots in Markov chain Monte Carlo (MCMC) iterations for the Chinese natural gas market (NG-China) and the international natural gas market (NG), the Chinese copper market (CU-China) and the international copper market (CU), and the Chinese aluminum market (AL-China) and the international aluminum market (AL).

Fig 3 illustrates the volatility characteristics of the natural gas, copper, and aluminum markets in China and globally during the MCMC iteration. As can be seen from Fig 3, the two chains mostly show random fluctuations and do not exhibit a clear evolutionary trend. With the passage of time and the increasing number of iterations, the trajectory line gradually smooths out. This phenomenon implies that the model has reached a state of convergence after long iterations. The convergence of the parameters verifies the validity of the model. With adequate iterations, the Bayesian method can effectively represent the volatility and correlation among the natural gas, copper, and aluminum markets internationally and within China.

**Fig 4:**
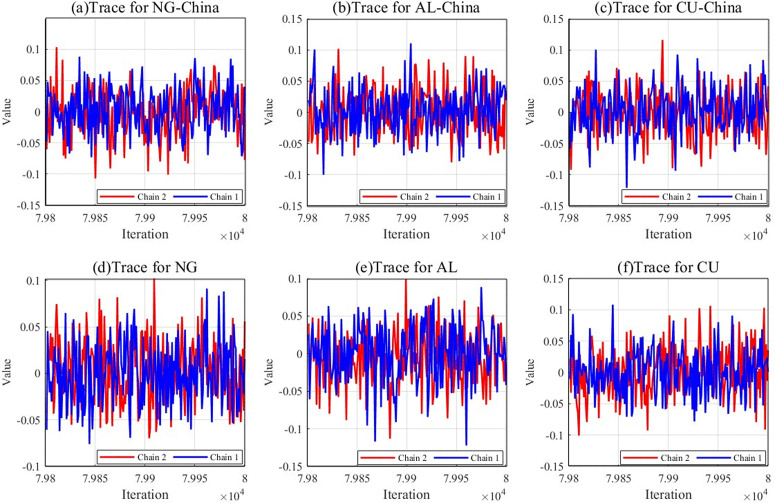
Tracking the dynamics of the international and chinese natural gas, copper, and aluminum markets.

Fig 4 illustrates the mutual fluctuations of two different chains between different iterations. As shown in Fig 4, the two chains fluctuate similarly and show a high degree of similarity in the fluctuation trajectories. At the same time, the two chains also fluctuate with each other in the process of operation. Such a phenomenon fully demonstrates that the MCMC algorithm has good hybridity, which enables the chains to show the charac-teristics of fit and good interaction in the operation process. At the same time, it suggests risk transmissibility among the international and Chinese markets for natural gas, copper, and aluminum. In addition, this mixture implies that the different Markov chains have fully exchanged information, thus ensuring the accuracy and stability of the posterior dis-tribution estimates. In other words, the parameter estimates are reliable, demonstrating the high applicability of the DGC-t-MSV model in studying volatility spillovers among in-ternational and Chinese natural gas, copper, and aluminum markets.

### 4.3 Spillover effect analysis of model mean

In Table 2, dynamic conditional correlation coefficients are applied to reflect mean spillover effects among the international and Chinese natural gas, copper, and aluminum markets. Mean spillover effects refer to the impact of price fluctuations in one market on prices in another market.

**Table 2:** Descriptive statistics of the dynamic correlation coefficients of the natural gas, copper, and aluminum markets indices for international and chinese markets.

Node	Mean	Std	Max	Min	Range
ρ NG(China)-AL(China)	0.00379	0.09965	0.23542	−0.31235	0.54777
ρ NG(China)-CU(China)	0.17251	0.15900	0.60690	−0.08177	0.68867
ρ NG(China)-AL	0.17414	0.12338	0.47156	−0.16442	0.63598
ρ NG(China)-CU	0.15896	0.11240	0.40113	−0.10340	0.50453
ρ NG-AL(China)	0.14716	0.15721	0.47115	−0.12355	0.59470
ρ NG-CU(China)	0.21136	0.09324	0.43775	−0.03722	0.47497
ρ NG-AL	0.14745	0.11341	0.39661	−0.06763	0.46424
ρ NG-CU	0.48998	0.09155	0.76107	0.30922	0.45185

Table 2 demonstrates the dynamic correlation coefficients among the global and Chinese natural gas, copper, and aluminum markets indices:

First, Dynamic correlations exist among the global natural gas, copper, and aluminum markets. For instance, the global natural gas market (NG) and global copper market (CU) show the strongest correlation, at 0.48998. A significant linkage also emerges between NG and the Chinese copper market (CU-China), with a coefficient of 0.21136. This can be partly attributed to the Russia-Ukraine war, which triggered international inflation and led to volatile turbulence in financial markets. The copper market is highly sensitive to such fluctuations. It possesses significant financial characteristics and plays a crucial role in trading, investment, and inflation hedging. When analyzed on an international scale, price fluctuations in the copper market tend to align closely with those observed in the natural gas market [33,34].

Second, price spillovers are more evident among the global natural gas, copper, and aluminum markets. For example, the Chinese natural gas market (NG-China) and the aluminum market in China (AL-China) exhibit a minimal correlation of 0.00379. In contrast, the international natural gas market (NG) and aluminum market (AL) show a stronger linkage at 0.14745. Furthermore, the relationship between global natural gas and copper markets is also higher than that between Chinese natural gas and copper markets. This can be attributed to the fact that the spillover relationship among Chinese natural gas, copper, and aluminum markets is highly politically sensitive, influenced by domestic demand, inventory changes, and policies [35,36].

**Fig 5:**
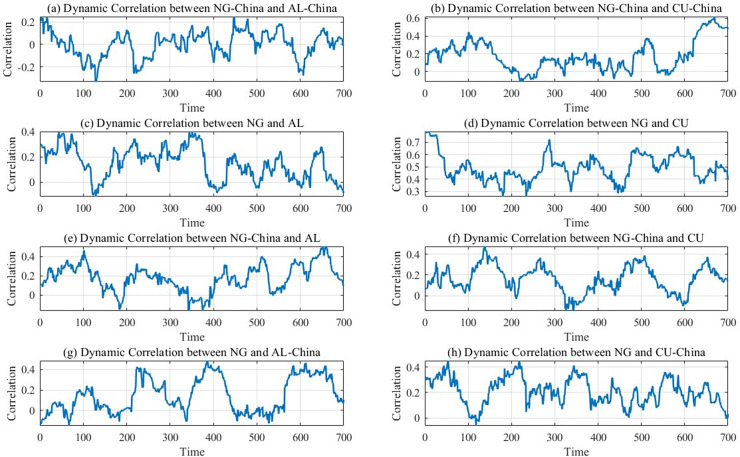
Correlation results of natural gas, copper, and aluminum market dynamics between international and Chinese markets.

From the specific analyses in Fig 5, it demonstrates that:

First, the spillovers among the international natural gas, copper, and aluminum markets are analyzed. Fig 5 (c) illustrates the dynamic relationship between the international natural gas and aluminum markets. The relevance coefficient fluctuates between 0 and 0.35, with a peak at node 350 before gradually decreasing. Fig 5 (d) shows the correlation between the international natural gas and copper markets. The coefficient oscillates between 0.3 and 0.7, declining from node 300, reaching its lowest point near node 350, and rebounding with multiple fluctuations. These patterns highlight the complexity of cross-market linkages and the stronger natural gas-copper correlation. This asymmetry stems from copper’s critical role as an international industrial material. Copper demand is tightly linked to macroeconomic health. Meanwhile, the Russia-Ukraine war disrupted international gas markets, triggering broader economic turbulence. These dual shocks amplified the interdependence between gas and copper markets [37].

Second, the analysis is conducted from the perspective of the Chinese natural gas, copper, and aluminum markets. Fig 5 (a) displays the dynamic correlation between the Chinese natural gas and aluminum markets, where the correlation coefficient fluctuates between −0.2 and 0.2, indicating a weak relationship. Fig 5 (b) illustrates the dynamic correlation between the Chinese natural gas and copper markets, with a positive correlation with fluctuations ranging from 0.2 to 0.6. The reason for this can be seen in the acceleration of the new Chinese energy grid and electric vehicle industry since 2023. Copper, as a key conductive material, enhances the interdependence between the Chinese natural gas and copper markets [38].

Third, an analysis of the interrelationship among the international and Chinese natural gas, copper, and aluminum markets is analyzed. Figs 5(e) and 5(f) depict dynamic correlations among Chinese natural gas, international copper and aluminum markets. Figs 5(g) and 5(h) present linkages among the international natural gas, Chinese copper and aluminum markets. Correlation coefficients across these pairings oscillate between 0 and 0.4. The weakest correlation occurs between the international natural gas and Chinese aluminum markets. Most coefficients remain below 0.2, indicating a weak relevance between the international natural gas and Chinese aluminum markets. The reason for this can be seen in the fact that although aluminum is an energy-intensive metal, in China, most of the aluminum production relies on coal power. Thus, the relationship between the international natural gas market and the Chinese aluminum market remains weak [35,39].

### 4.4 Analysis of price spillover effects of the model

This part investigates the lead-lag relationship and volatility transmission direction among the Chinese and international natural gas markets, as well as the Chinese and international copper and aluminum markets, which will be reflected by volatility spillover effects. Table 3 presents the parameter estimation results for the volatility levels of the six indices. The analysis emphasizes the mean and standard deviation of their volatility, along with their distributional characteristics within the model. This information provides a comprehensive insight into the fluctuations of specific markets, which can be seen from the specific analyses in Table 3:

**Table 3:** Volatility level parameters for natural gas, copper, and aluminum markets between international and Chinese markets.

Node	Mean	Standard Deviation	MCError	2.50%	5.00%	Median	97.50%
μNG-China	2.26001	3.61253	0.12012	0.00459	0.01182	0.86518	13.50711
μAL-China	2.40668	4.38055	0.12298	0.00291	0.00965	0.64075	15.66588
μCU-China	2.05696	3.02938	0.11107	0.00503	0.01391	0.98448	12.14950
μNG	2.17644	4.00936	0.11136	0.00067	0.00534	0.75937	14.08174
μAL	2.45462	4.35891	0.12991	0.00351	0.00781	0.92029	14.18173
μCU	2.21399	3.83033	0.11510	0.00133	0.00477	0.73767	13.68160

First, the volatility levels of both the international and Chinese aluminum markets are higher than the other indices. For example, the volatility level parameter is 2.45462, which represents the international aluminum market. While the standard value is 4.35891, indicating that the aluminum market is highly volatile. The reason for this can be seen in the price volatility in the aluminum market, which may be related to the Russian-Ukrainian conflict during the study period. The Russia-Ukraine conflict, which are major gas storage countries, resulted in instability in the gas market, heightening the risk of supply disruptions or constraints. The rise in natural gas prices, in turn, increased the cost of aluminum production, thus generating sharp fluctuations in the aluminum market as well [40].

Second, the volatility parameter for China’s copper market (CU-China) is 2.05696. The standard deviation is 3.02938, making it the lowest among all markets. This indicates relatively low volatility in the Chinese copper market. Such stability is closely linked to China’s economic policies and market regulations. Copper holds significant strategic importance for China, and the country has implemented targeted policies to secure its supply chain. Therefore, China’s copper market is highly politically sensitive, limiting the impact of international policies and geopolitical conflicts.

The volatility persistence parameter is often used to quantify the time dependence of asset price fluctuations, that is, to measure the correlation between current and past volatility. Table 4 measures the two-way transmission relationship among the international and Chinese natural gas, copper, and aluminum markets through the volatility spillover effect parameter (φ). The specific analyses are presented in Table 4.

**Table 4:** Table of Simulation results for spillovers in price volatility between international and Chinese markets for natural gas, copper, and aluminum.

Node	Mean	SD	MC Error	2.50%	5.00%	Median	97.50%
φ AL-China to NG-China	−0.00201	0.00269	0.00190	−0.00456	−0.00443	−0.00201	0.00055
φ NG-China to AL-China	0.00349	0.00467	0.00331	−0.00095	−0.00071	0.00349	0.00793
φ CU-China to NG-China	−0.00040	0.00006	0.00004	−0.00046	−0.00046	−0.00040	−0.00034
φ NG-China to CU-China	−0.00054	0.00066	0.00046	−0.00116	−0.00113	−0.00054	0.00009
φ AL to NG-China	−0.00035	0.00046	0.00033	−0.00079	−0.00076	−0.00035	0.00009
φ NG-China to AL	−0.00066	0.00053	0.00038	−0.00116	−0.00114	−0.00066	−0.00015
φ CU to NG-China	−0.00004	0.00008	0.00006	−0.00011	−0.00011	−0.00004	0.00004
φ NG-China to CU	−0.00002	0.00117	0.00083	−0.00113	−0.00107	−0.00002	0.00110
φ AL-China to NG	−0.00083	0.00402	0.00284	−0.00465	−0.00445	−0.00083	0.00299
φ NG to AL-China	0.00126	0.00059	0.00042	0.00070	0.00073	0.00126	0.00182
φ CU-China to NG	0.00407	0.00452	0.00319	−0.00023	0.00000	0.00407	0.00836
φ NG to CU-China	0.00071	0.00015	0.00010	0.00057	0.00058	0.00071	0.00085
φ AL to NG	0.00509	0.00588	0.00416	−0.00050	−0.00021	0.00509	0.01068
φ NG to AL	0.00068	0.00039	0.00027	0.00031	0.00033	0.00068	0.00105
φ CU to NG	0.00237	0.00233	0.00165	0.00016	0.00027	0.00237	0.00458
φ NG to CU	0.00033	0.00035	0.00024	0.00000	0.00001	0.00033	0.00065

First, the two-way spillover effect between China’s aluminum market (AL-China) and China’s natural gas market (NG-China) is positive and negative. Among them, the aluminum market in China has a negative spillover to the natural gas market in China, with a value of −0.00201. In contrast, the natural gas market in China exhibits a positive spillover effect on the aluminum market, with a value of 0.00349. This indicates that fluctuations in the Chinese natural gas market influence aluminum prices. The impact occurs through the price transmission mechanism. Meanwhile, the negative spillover implies that the Chinese aluminum market may provide hedging and stabilizing effects for the Chinese natural gas market [41]. A similar asymmetric pattern is also found in the bidirectional volatility spillovers between the Chinese aluminum market and the international natural gas market, with one direction negative and the other positive. Taken together, these results suggest that the aluminum market functions mainly as a risk absorber in risk spillovers. [42,43]. Because aluminum production is highly energy-intensive, volatility shocks from the natural gas market are more readily transmitted into aluminum costs and prices, making the aluminum market more likely to act as a net receiver of risk in the spillover network.

Second, the two-way spillovers between China’s copper market (CU-China) and China’s natural gas market (NG-China) are negative, with spillover values of −0.00040 and −0.00054. This indicates that when volatility in the Chinese copper market increases, volatility in the Chinese natural gas market decreases, and vice versa. Similarly, the two-way spillover effects between the international copper market (CU) and the Chinese natural gas market (NG-China) are also negative. From a portfolio perspective, this negative spillover relationship indicates potential hedging effects between these markets. However, the spillovers between China’s copper market and natural gas market are insignificant, while there is little link between the global copper market and China’s natural gas market, which implies that China’s natural gas market has a minor impact on the copper market. The reasons involve the considerable political sensitivity of China’s natural gas sector and its significant reliance on natural gas imports. Consequently, the Chinese natural gas market exerts less influence on both the international and Chinese copper markets [44–46].

Third, the price spillovers between the international aluminum market (AL) and the international natural gas market (NG), as well as the spillovers between the international copper market (CU) and the international natural gas market (NG) are all positive, that spillover values are close to the significant level. However, there is a more notable influence of price fluctuations between the international markets for natural gas and aluminum. The reason for this can be seen in the fact that during the study period, the Russian-Ukrainian conflict led to a surge in natural gas prices, which directly pushed up the cost of aluminum production. As for copper, as an international industrial metal, the international copper market is influenced not only by the impact of the energy market but also by macroeconomic and market capital flows. As a result, the spillover effects between the international gas and aluminum markets are more substantial [47,48].

### 4.5 Test analysis of the model

Model convergence analysis ensures that the model effectively converges to the optimal solution within a reasonable time frame, thus avoiding unnecessary processes. This paper focuses on evaluating whether the model parameters or loss function values show a gradual stabilization trend as the number of iteration rounds increases during the execution of the optimization algorithm or the model’s training to determine whether the model has reached the desired ideal performance state.

**Fig 6:**
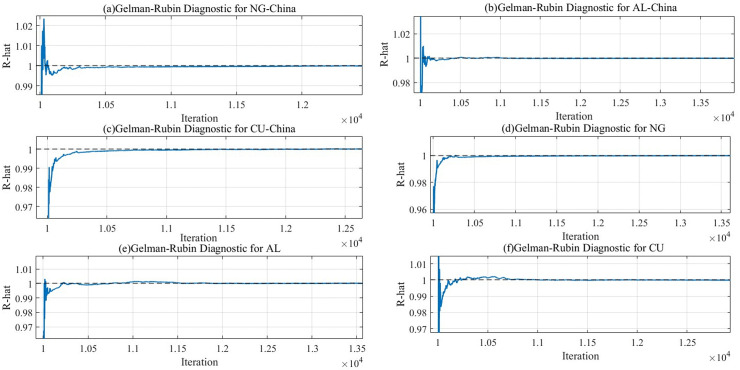
Gelman-Rubin test results.

The Gelman-Rubin test is a commonly used method to evaluate the convergence of MCMC (Markov Chain Monte Carlo) sampling. By comparing the SRF values of different chains, if the value tends to 1, sufficient mixing has been achieved between the chains. That is, the sampling process has reached a state of convergence. The methodology entails rigorous evaluation of convergence patterns across MCMC chains, aiming to confirm the stability of the parameter estimates in the Bayesian analysis and verify the reasonableness of the posterior distribution of the parameters, thus ensuring that the analysis results are accurate and reliable. Fig 6 illustrates the Gelman-Rubin test results:

During initial iterations, the shrinkage factor exhibits pronounced magnitude, which progressively diminishes and asymptotically stabilizes with continued algorithmic progression, signifying that the posterior distribution has reached a steady state. The accelerated convergence profile confirms the stability of model parameters’ posterior distribution, further indicating the convergence of the MCMC chain. This result also verifies that the DGC-t-MSV model is parametrically stable and reliable in analyzing the spillover effects among the natural gas, copper, and aluminum markets in international and Chinese contexts. At the same time, after sufficient iteration, the Bayesian approach can accurately capture the complex dynamic linkages among the international and Chinese natural gas, copper, and aluminum markets.

## 5 Robustness checks

### 5.1 Robustness to USD exchange rate movements

**Fig 7:**
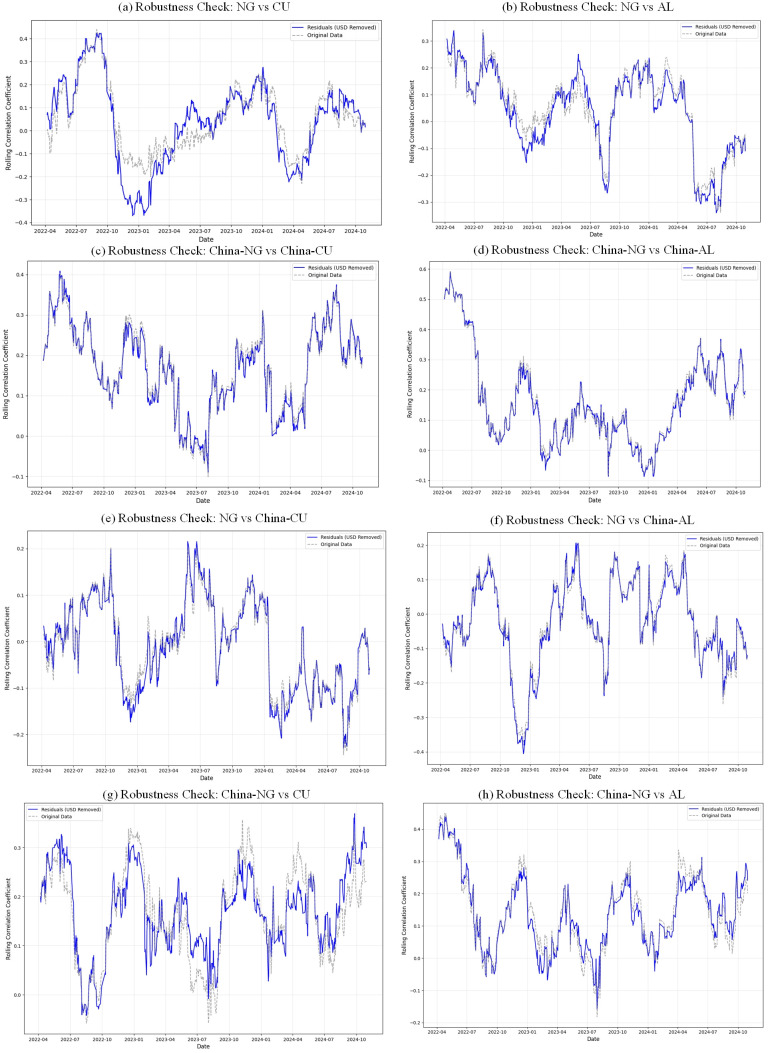
Comparison of Dynamic Correlations before and after Controlling for USD Index Influence.

To examine whether movements in the US dollar exchange rate materially affect the correlations between natural gas and copper and aluminum markets, this subsection conducts a robustness check. A two-step orthogonalization procedure is employed. First, the log returns of all natural gas, copper, and aluminum price series are regressed on the log return of the US Dollar Index (DXY), and the regression residuals $\varepsilon_{i,t}$ are taken as returns net of the linear USD effect. Second, 60-day rolling correlations are computed for these residual series and compared with the rolling correlations based on the original return data. The results are reported in Fig 7, where solid lines represent the USD-adjusted residual correlations and dashed lines represent the original correlations.

Fig 7(a)–7(h) displays the time-varying correlations between international and Chinese natural gas and copper and aluminum markets, before and after controlling for the USD index. The USD-adjusted correlation paths are generally close to their baseline counterparts, with similar timing of peaks, troughs, and sign changes and only moderate differences in levels; for the pairs involving Chinese markets, the two sets of correlation paths almost coincide. Overall, this robustness check suggests that the main time-varying comovements between natural gas and copper and aluminum markets are not driven by omitted USD exchange rate effects.

### 5.2 Robustness to proxy replacement and sample extension

**Fig 8:**
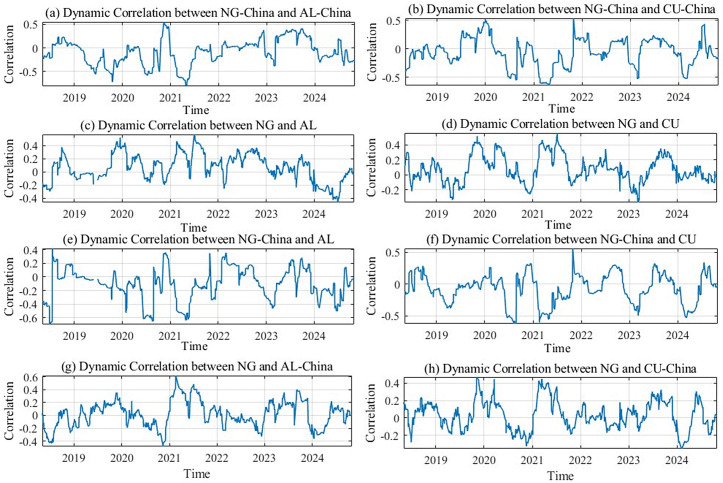
Robustness check: Dynamic correlations.

To examine the sensitivity of our parameter estimates to proxy selection, the article further conduct an alternative-measure robustness check by replacing China New Natural Gas stock price, which is used in the original setting as a proxy for the China natural gas market, with China LNG spot price data, and by extending the sample from 1 January 2022–31 October 2024–1 January 2018–31 October 2024. Holding the model structure and estimation procedure unchanged, the article re-estimate the DGC-t-MSV model, report the corresponding volatility-level parameter estimates in Table 5, and present the dynamic-correlation results in Fig 8.

**Table 5:** Robustness check: Volatility-level parameters.

Node	Mean	Standard Deviation	MCError	2.50%	5.00%	Median	97.50%
μNG-China	2.95407	4.96100	0.34340	0.00736	0.01363	1.13497	22.25880
μAL-China	2.14310	3.21490	0.08249	0.00000	0.00002	0.90388	12.19795
μCU-China	2.38024	3.46883	0.09336	0.00001	0.00197	1.13196	12.28857
μNG	2.59123	4.24726	0.10324	0.00116	0.00510	0.85679	14.69283
μAL	2.70468	4.55518	0.12048	0.00059	0.00824	1.01782	16.66008
μCU	2.74377	4.53175	0.10672	0.00294	0.01005	1.00681	15.58700

The robustness results indicate that, using the extended-sample China LNG spot price data, the posterior estimates of the volatility-level parameters remain broadly stable, and the dynamic correlations continue to exhibit pronounced time variation with comparable fluctuation ranges; the key episode patterns are also broadly consistent with those obtained under the original setting. The supporting evidence is reported in Table 5 and Fig 8. Overall, the main inferences on the co-movement among natural gas, copper and aluminum markets are not driven by the choice of the China-market proxy, supporting the robustness of the findings.

### 5.3 Robustness to comparison of model specifications

To assess the robustness of our empirical findings and further justify the choice of the DGC-t-MSV specification, we benchmark it against the Gaussian DCC-MSV model. This comparison examines whether allowing for Student-t innovations and a more flexible time-varying dependence structure leads to material improvements in both in-sample fit and out-of-sample density forecasting. We employ two complementary metrics: the Akaike Information Criterion (AIC) for in-sample goodness of fit and the Log Predictive Score (LPS) for out-of-sample density forecasting accuracy.

Table 6 shows that the DGC-t-MSV model yields an AIC of 12,844.12, which is lower than the DCC-MSV benchmark’s 13,205.84. This indicates that DGC-t-MSV fits the in-sample data better even after taking into account the need to avoid overly complicated models that may overfit the sample. In addition, Table X reports an LPS of −1,715.33 for DGC-t-MSV, which is higher than the DCC-MSV value of −1,842.15 and thus closer to zero, suggesting more accurate out-of-sample predictive densities. Overall, these results support the appropriateness of the DGC-t-MSV specification and indicate that the main results derived from it are robust.

**Table 6:** Robustness check: Model comparison results.

Model	LPS (Out-of-Sample)	AIC (In-Sample)
DGC-MSV	−1,842.15	13,205.84
DGC-t-MSV	−1,715.33	12,844.12

## 6 Conclusion

This paper adopts the DGC-t-MSV model, combines Markov chain Monte Carlo and Bayesian methods for the model’s parameter estimation, and selects representative price data in the international and Chinese natural gas, copper, and aluminum markets to analyze the price spillover effects among the natural gas, copper, and aluminum markets. Key findings from this study are as follows:

(1) There are two-way price spillover effects among the international and Chinese natural gas, copper, and aluminum markets. Price spillover effects are stronger among the international natural gas, copper, and aluminum markets than between their Chinese counterparts. Dynamic spillovers between international and Chinese markets exhibit time-varying characteristics. These spillover effects fluctuate due to external factors such as policy changes and geopolitical events. They also display asymmetries across different contexts.
(2) Price spillover effects are more evident between the international and Chinese natural gas markets and the aluminum markets, with a stronger correlation observed between the international and Chinese natural gas and copper markets. The spillovers from the international and Chinese natural gas markets to the copper and aluminum markets are more substantial than the opposite direction, which indicates that the international and Chinese copper and aluminum markets act as risk recipients.
(3) The spillover effects between the international natural gas market and China’s copper-aluminum markets are more significant. Among them, compared with the spillover effects between the international natural gas market and the Chinese copper market, the spillovers between the international natural gas market and the Chinese aluminum market are more significant. The spillover effects between China’s natural gas market and the international copper-aluminum markets are not significant. The international and Chinese natural gas and copper-aluminum markets are mature markets with low volatility. Under extreme risks, the spillovers are more significant, which occurs between international and China’s natural gas and copper-aluminum markets.

Based on the empirical findings regarding price spillovers and co-movements between the natural gas and copper-aluminum markets, policy initiatives should prioritize two strategic fronts: early warning mechanisms and risk hedging capabilities. First, regulators and exchanges should establish a comprehensive cross-market monitoring system that tracks rolling spillover intensity, time-varying correlations, and tail-risk volatility using a tiered early-warning framework. Upon triggering specific risk thresholds, coordinated interventions, such as enhanced information disclosure, systematic stress testing, and the dynamic adjustment of margin requirements, should be implemented to curb risk contagion across the energy-metal nexus. Given that the 2022–2024 sample largely falls within a heightened geopolitical “crisis regime” associated with the Russia–Ukraine conflict, early-warning thresholds and related interventions should be designed in a state-contingent manner, as spillover strength and transmission channels may differ in more stable periods. Second, policymakers should facilitate a robust risk-management toolkit for enterprises by deepening the derivatives market for these commodities and issuing practical hedging guidelines. Concurrently, firms should be encouraged to synergize contract design, inventory management, and hedging strategies to stabilize input costs, thereby mitigating exposure to cross-market fluctuations and enhancing supply-chain resilience.

The empirical analysis in this paper enriches the study of volatility spillovers among the natural gas, copper, and aluminum markets. It helps to provide a basis for forecasting the price trends in the international natural gas, copper, and aluminum markets. However, during the study, it was found that China has yet to establish a formal natural gas futures market and that the volatility of the copper and aluminum markets is affected by several factors. For example, investor sentiment in the copper and aluminum markets may amplify the impact on the natural gas market. Therefore, future research will incorporate text analysis, social media data, and other market sentiment analysis to further research the price spillovers among the natural gas, copper, and aluminum markets.
